# Erratic Pulse Oximetry Pulse Rate With Preserved Oxygen Saturation in a Patient Wearing White Nail Polish: A Case Report

**DOI:** 10.7759/cureus.101995

**Published:** 2026-01-21

**Authors:** Akhtar Purvez

**Affiliations:** 1 Clinical Research, Momentum Medical Research, Charlottesville, USA; 2 Clinical Sciences, Lincoln Memorial University DeBusk College of Osteopathic Medicine, Harrogate, USA

**Keywords:** arrhythmia mimic, diagnostic error, nail polish, patient safety, photoplethysmography, pulse oximetry

## Abstract

Pulse oximetry is widely used for noninvasive monitoring of oxygen saturation (SpO₂) and pulse rate. Because the pulse rate displayed by a pulse oximeter is derived from the peripheral photoplethysmographic waveform, it can be affected by artifact even when oxygen saturation appears normal. We report a case of an asymptomatic patient wearing white nail polish who demonstrated stable, normal SpO₂ values yet markedly erratic pulse rate readings on pulse oximetry, fluctuating rapidly between bradycardic and tachycardic ranges. Concern for arrhythmia prompted a 12‑lead electrocardiogram, which showed a normal sinus rhythm. This report highlights a practical diagnostic pitfall: nail cosmetics may have little effect on SpO₂ yet still destabilize pulse waveform detection and generate misleading pulse rate values, leading to unnecessary evaluation and erroneous clinical conclusions.

## Introduction

Pulse oximetry estimates arterial oxygen saturation using differential absorption of red and infrared light, and it derives pulse rate from the periodicity of the photoplethysmographic (PPG) waveform rather than from direct cardiac electrical activity. As a result, the displayed pulse rate may diverge from the true heart rate when the PPG signal is degraded by motion, poor perfusion, ambient light, or optical interference [1-3]. Importantly, pulse oximetry does not assess cardiac electrical rhythm and should not be used to diagnose arrhythmias; abnormal pulse rate readings reflect peripheral waveform detection rather than intrinsic cardiac conduction. While much of the existing literature has focused on factors affecting oxygen saturation (SpO₂) accuracy, less attention has been given to scenarios in which artifacts selectively impair pulse rate reliability despite preserved SpO₂, which is the central diagnostic limitation highlighted by this case. Clinicians frequently interpret the pulse oximeter pulse rate as synonymous with heart rate, especially in time-pressured environments, which can contribute to diagnostic errors when readings are artifactually abnormal [2,3].

## Case presentation

A female patient presented for routine clinical evaluation. The patient was an adult female who was hemodynamically stable and asymptomatic, without known comorbidities or medications affecting peripheral perfusion. She denied dizziness, palpitations, chest pain, or dyspnea. Pulse oximetry was performed using a standard fingertip pulse oximeter in the clinical setting. It displayed oxygen saturation values within the normal range, without notable variability. In contrast, the displayed pulse rate was highly erratic, fluctuating over seconds between approximately 40 beats per minute, 50 beats per minute, and values exceeding 100 beats per minute. The pulse rate fluctuated over seconds between approximately 40-50 beats per minute and values exceeding 100 beats per minute, prompting electrocardiographic evaluation during the same clinical encounter. Pulse oximetry was repeated after removal of the white nail polish from the monitored finger, after which pulse rate readings stabilized and correlated with the electrocardiographic heart rate. However, concerned about an underlying rhythm disturbance, a 12‑lead electrocardiogram (ECG) was obtained and demonstrated a normal sinus rhythm with a stable rate and no conduction abnormality. Figure 1 illustrates a normal sinus rhythm.

**Figure 1:**
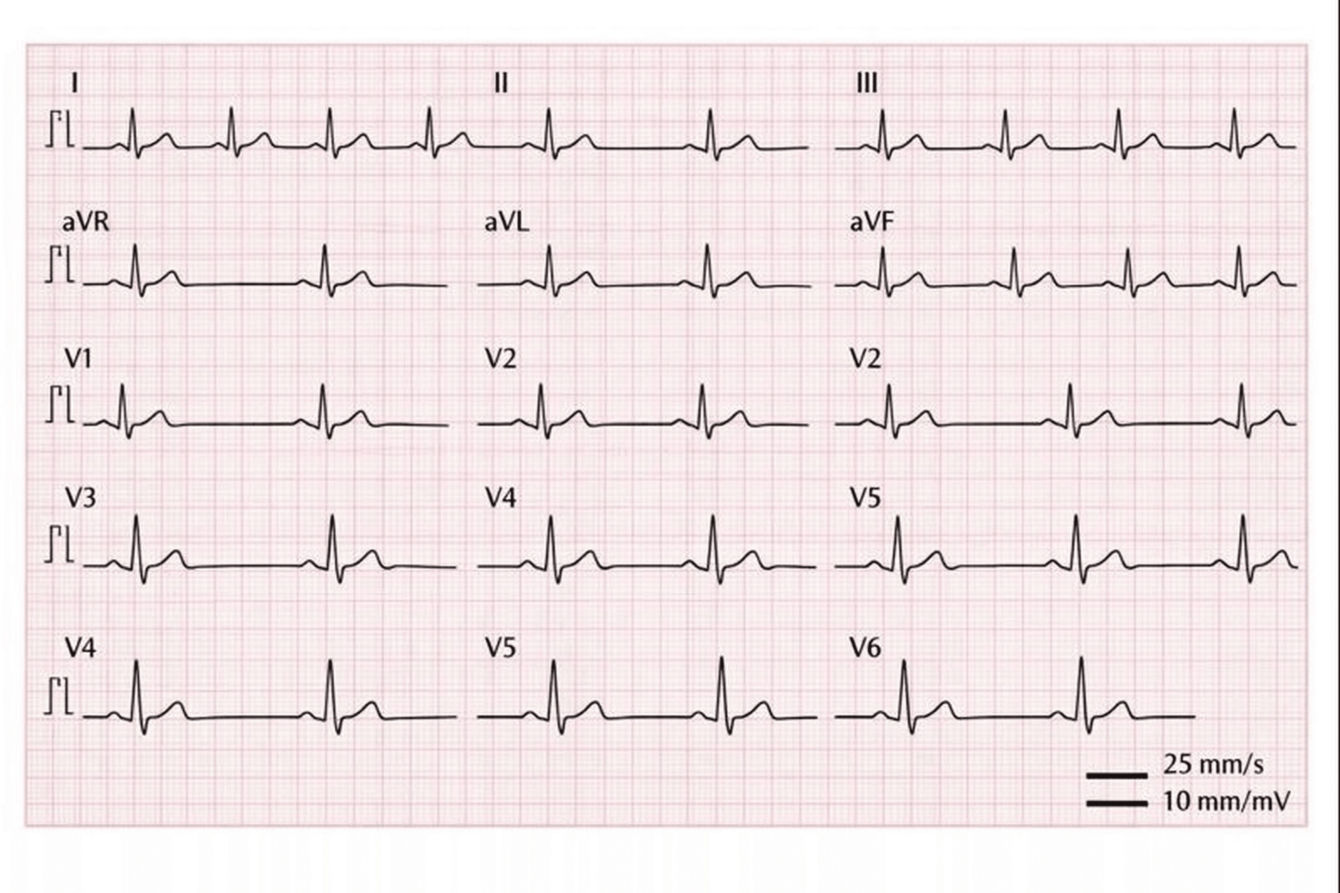
EKG showing normal sinus rhythm

A physical examination showed that the finger used for pulse oximetry had white nail polish on it. The discordance between normal ECG findings and the rapidly changing oximeter pulse rate suggested an artifactual PPG signal rather than a true arrhythmia.

## Discussion

This case demonstrates a clinically important dissociation: stable, normal SpO₂ readings occurred alongside a markedly unstable pulse rate display. Because pulse rate is derived from the photoplethysmographic waveform, any factor (Table 1) that reduces waveform fidelity can produce spurious pulse detection while SpO₂ remains largely unaffected due to internal averaging and filtering mechanisms [1,3]. The resulting monitor output may mimic bradyarrhythmia, tachyarrhythmia, or an irregular pulse pattern, potentially triggering unnecessary electrocardiography, monitoring escalation, or cardiology consultation [2]. Importantly, pulse oximetry does not assess cardiac electrical rhythm, and abnormal pulse rate readings derived from photoplethysmography should prompt confirmatory evaluation rather than be interpreted as diagnostic of true arrhythmia.

**Table 1:** Common Causes of Pulse Oximetry Discrepancy

Cause	Typical Mechanism
Motion artifact	Distorts PPG waveform and pulse detection; may variably affect SpO₂ and pulse rate [1, 3].
Poor peripheral perfusion (e.g., vasoconstriction, shock)	Reduces pulsatile signal amplitude; increases noise-to-signal ratio [1,3].
Ambient light intrusion	Adds optical noise to the sensor signal [1,3].
Nail cosmetics (polish, gel, acrylics)	Alters light transmission/reflection; may destabilize waveform detection [4-7].
Skin pigmentation / dyshemoglobins	Systematic bias in SpO₂ under some conditions may complicate interpretation [8].

Nail polish and other nail cosmetics are well studied as sources of optical interference in pulse oximetry. Notably, most prior literature on nail cosmetics and pulse oximetry has focused on the accuracy of oxygen saturation (SpO₂) measurements, with relatively limited attention to the reliability of pulse rate derived from photoplethysmographic waveforms. Pulse oximeters emit red (~660 nm) and infrared (~940 nm) light through the digit and infer oxygen saturation from differential absorption by oxygenated and deoxygenated hemoglobin [1, 2]. Pigments applied to the nail plate can alter light transmission and reflection, particularly darker shades such as black, brown, and dark blue, which may absorb incident light and increase measurement variability [4-6]. Even when statistically detectable changes occur, most studies report oxygen saturation errors of less than 2%, remaining within manufacturer performance specifications [6,7].

Studies have shown that white or clear nail polish has a minimal effect on SpO₂ measurements that are clinically important [4-7]. White pigments tend to reflect the wavelengths used in pulse oximetry instead of absorbing them. Clear polish has very little pigment in it. Accordingly, removal of dark nail polish is typically recommended when precise oxygenation assessment is critical, whereas white or clear polish is generally considered acceptable for routine SpO₂ monitoring [6,7]. However, this case highlights that preservation of SpO₂ accuracy does not guarantee pulse rate reliability, as photoplethysmographic waveform instability may still result in erratic pulse detection.

Prior studies evaluating the effect of nail polish on pulse oximetry have largely focused on oxygen saturation (SpO₂) accuracy and have generally concluded that white or clear nail polish produces minimal clinically meaningful error. However, these studies have placed comparatively less emphasis on the reliability of pulse rate derived from photoplethysmographic waveforms. In this case, preservation of SpO₂ accuracy alongside erratic pulse rate readings suggests that white nail polish may interfere with waveform stability or peak detection without significantly altering averaged saturation values, reflecting differences in signal processing for SpO₂ versus pulse rate. From a patient safety perspective, this dissociation is clinically important, as reliance on artifact-prone pulse rate readings may prompt unnecessary electrocardiography, monitoring escalation, or specialist consultation. Increased awareness of this limitation may help reduce diagnostic error, patient anxiety, and avoidable healthcare resource utilization.

For bedside practice, abnormal pulse oximeter pulse rates should be correlated with palpated pulse, auscultation, or electrocardiography when the finding would influence clinical management [2, 3]. Signal quality can often be improved by minimizing motion, optimizing peripheral perfusion, shielding ambient light, repositioning the sensor, or selecting an alternative measurement site [1-3]. In settings requiring maximal accuracy, particularly during hypoxemia or low-perfusion states, removal of nail polish - especially dark colors - or use of a different probe location remains a prudent precaution [6, 7, 9, 10].

## Conclusions

White nail polish is unlikely to cause clinically meaningful error in pulse oximetry-derived oxygen saturation (SpO₂) measurements in most clinical settings. However, as demonstrated in this case, even when SpO₂ values remain stable and within the normal range, white nail polish may still destabilize photoplethysmographic (PPG) waveform detection and produce markedly erratic pulse rate readings. This dissociation reflects the fundamentally different signal processing pathways used to derive oxygen saturation versus pulse rate.

Recognition that the pulse rate displayed by pulse oximetry is an indirect, artifact-prone measurement - rather than a direct reflection of cardiac electrical activity - is essential for accurate clinical interpretation. Failure to appreciate this distinction may lead to erroneous conclusions, unnecessary diagnostic testing, and avoidable patient anxiety. A careful correlation of pulse oximeter findings with clinical assessment, palpated pulse, and electrocardiography, when appropriate, can mitigate diagnostic errors and prevent unwarranted cardiac evaluation. When pulse oximetry pulse rate readings are abrupt, highly variable, or discordant with clinical assessment or oxygen saturation, heart rate should be verified by palpation or electrocardiography before attributing findings to true arrhythmia.
